# Case Report: Gastric cancer with chondromyxoid matrix similar matrix-producing metaplastic breast carcinoma: report of an undescribed entity

**DOI:** 10.3389/fonc.2023.1087241

**Published:** 2023-05-19

**Authors:** Xiaoyan Wu, Huihua He, Lina Zhao, Zhi Zeng, Jingping Yuan

**Affiliations:** ^1^ Department of Pathology, Renmin Hospital of Wuhan University, Wuhan, China; ^2^ Department of Pathology, West China Hospital, Sichuan University, Chengdu, China

**Keywords:** gastric cancer, chondromyxoid matrix, matrix-producing metaplastic carcinoma, adenocarcinoma, histologic morphology, case report

## Abstract

Gastric cancer is a malignant epithelial neoplasm of the stomach, including adenocarcinoma, squamous cell carcinoma, adenosquamous carcinoma, undifferentiated carcinoma, gastroblastoma and neuroendocrine neoplasms, without gastric metaplastic carcinoma. We describe a 69-year-old male patient with gastric cancer who presented with a novel, biphasic histologic morphology with one component consisting of poorly differentiated adenocarcinoma and the other component consisting of chondromyxoid matrix with adenocarcinoma transition to, between the two components without a spindle cell component. The histological morphology of this case is similar to matrix-producing metaplastic breast carcinoma. Therefore, we diagnose this case as gastric carcinoma with chondromyxoid matrix similar matrix-producing metaplastic breast carcinoma.

## Introduction

Gastric carcinoma with chondromyxoid matrix similar matrix-producing metaplastic breast carcinoma is the first reported, which is a malignant epithelial tumor with heterologous mesenchymal differentiation. Our case is a carcinoma with a direct transition from adenocarcinoma to cartilaginous matrix lacking an intervening spindle cell component. This case is similar to metaplastic breast carcinoma with matrix-producing, which is characterized by a direct transition from invasive breast carcinoma no special type (IBC-NST) to cartilaginous/osseous matrix without an interspersed spindle cell component (1). We report this case because of its specific morphological features, which need to be described.

## Case presentation

**Figure 1:**
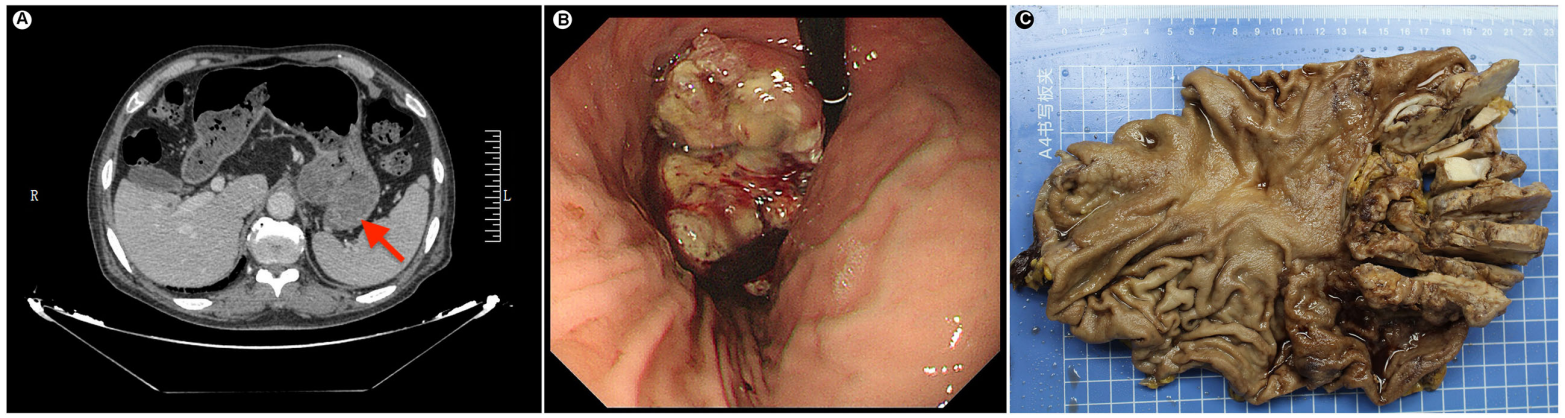
**(A)** Preoperative radiology image shows a large raised mass at the fundus-cardia (red arrow). **(B)** Gastric endoscopy shows a large raised mass at the fundus-cardia. **(C)** Gross photograph of the gastric specimen. The tumor section shows gray-white, slightly hard and relatively clear borders.

A 69-year-old male patient presented with a gastric mass for one week. A gastric mass was found by gastric endoscopy in another hospital, and biopsy was performed. The pathological results showed chronic inflammation of the stomach, and no tumor cells were found. The patient’s serum tumor marker CA72-4 was 433 IU/mL. For a clear diagnosis, abdominal CT and gastric endoscopy were performed again. A large mass was seen at the stomach fundus and cardia, with a central depression and erosion (**Figures 1A, B**). The rebiopsy results showed a malignant tumor of the stomach fundus and cardia, with a high possibility of adenocarcinoma. Furthermore, the patient had a history of hyperuricemia for many years and had taken colchicine orally for a long time. He also had grade 3 hypertension for more than 2 years and was treated with oral amlodipine besylate tablets. No smoking and no alcohol drinking.

**Figure 2:**
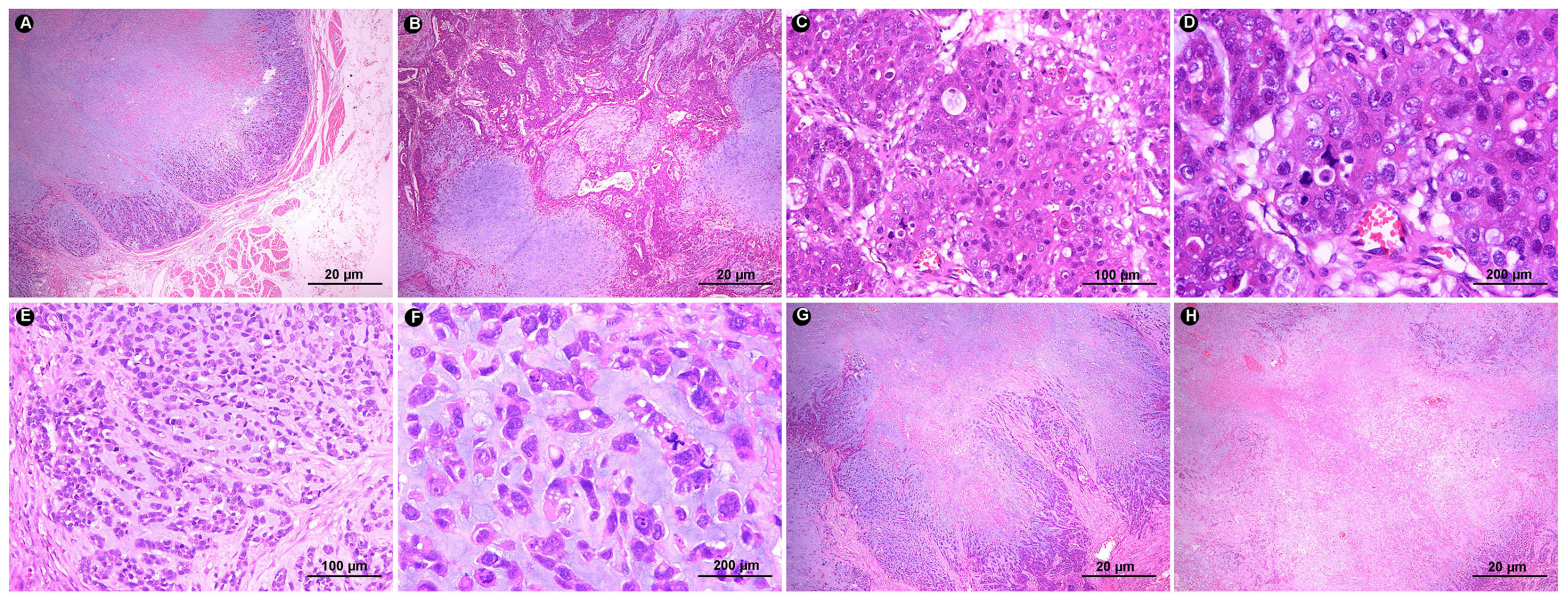
**(A)** In low power, the tumor border is relatively clear and shows expansile infiltration (HE ×20). **(B)** The tumor has two components: adenocarcinoma area and chondromyxoid matrix area. The chondromyxoid matrix exhibits multinodular growth with direct transition of adenocarcinoma components (HE ×20). **(C)** Tumor cells in the carcinomatous area exhibit solid and cribriform morphology (HE ×100). **(D)** Tumor cells show rich eosinophilic cytoplasm, obvious eosinophilic nucleoli and pathological mitosis (HE ×200). **(E)** In the chondromyxoid area, tumor cells are distributed in the form of beams, cords, and single cells within it (HE ×100). **(F)** Tumor cells are eosinophilic, with obvious nuclear atypia, prominent nucleoli, and easy-to-see pathological mitoses (HE ×200). **(G)** The chondromyxoid area is multinodular, with abundant tumor cells around the nodules and sparse tumor cells in the middle (HE ×20). **(H)** Hemorrhage and necrosis are seen in the larger nodules (HE ×20).

Based on rebiopsy results, an open total gastrectomy was performed, which lasted approximately 4 hours with intraoperative bleeding of 100 ml. Gross examination showed a gray-white raised tumor with a size of approximately 10 cm×6.5 cm×4 cm at the stomach fundus and cardia (**Figure 1C**). The tumor section was relatively well circumscribed, gray-white and lightly hard. On microscopic examination, the tumor showed expansile infiltration with relatively well-circumscribed in low power (**Figure 2A**). Two distinct types of areas were seen within the tumor, including poorly differentiated adenocarcinoma area and area rich in chondromyxoid matrix, and the carcinoma transitions directly to chondromyxoid matrix (without spindle cell sarcomatoid component and osteoclast giant cell differentiation) (**Figure 2B**). The tumor cells in the adenocarcinoma area were arranged in a solid and cribriform pattern, with eosinophilic cytoplasm and large nuclei (**Figure 2C**). Eosinophilic nucleoli are obvious in high power, and pathological mitoses were easy to see (**Figure 2D**). The chondromyxoid matrix was widely distributed in a multinodular pattern, interspersed with poorly differentiated adenocarcinoma and directly migrating to it (**Figure 2B**). Tumor cells were trabecular, cord-like and single cells distributed in the chondromyxoid matrix (**Figure 2E**), with large nuclei, prominent eosinophilic nucleoli and obvious pathological mitoses (**Figure 2F**). The periphery of the nodules were more cellular with gradually diminishing cellularity toward the central area (**Figure 2G**). Hemorrhage and necrosis were present in the center of larger nodules (**Figure 2H**). Adenocarcinoma metastases were seen in 7 out of 21 lesser curvature lymph nodes.

**Figure 3:**
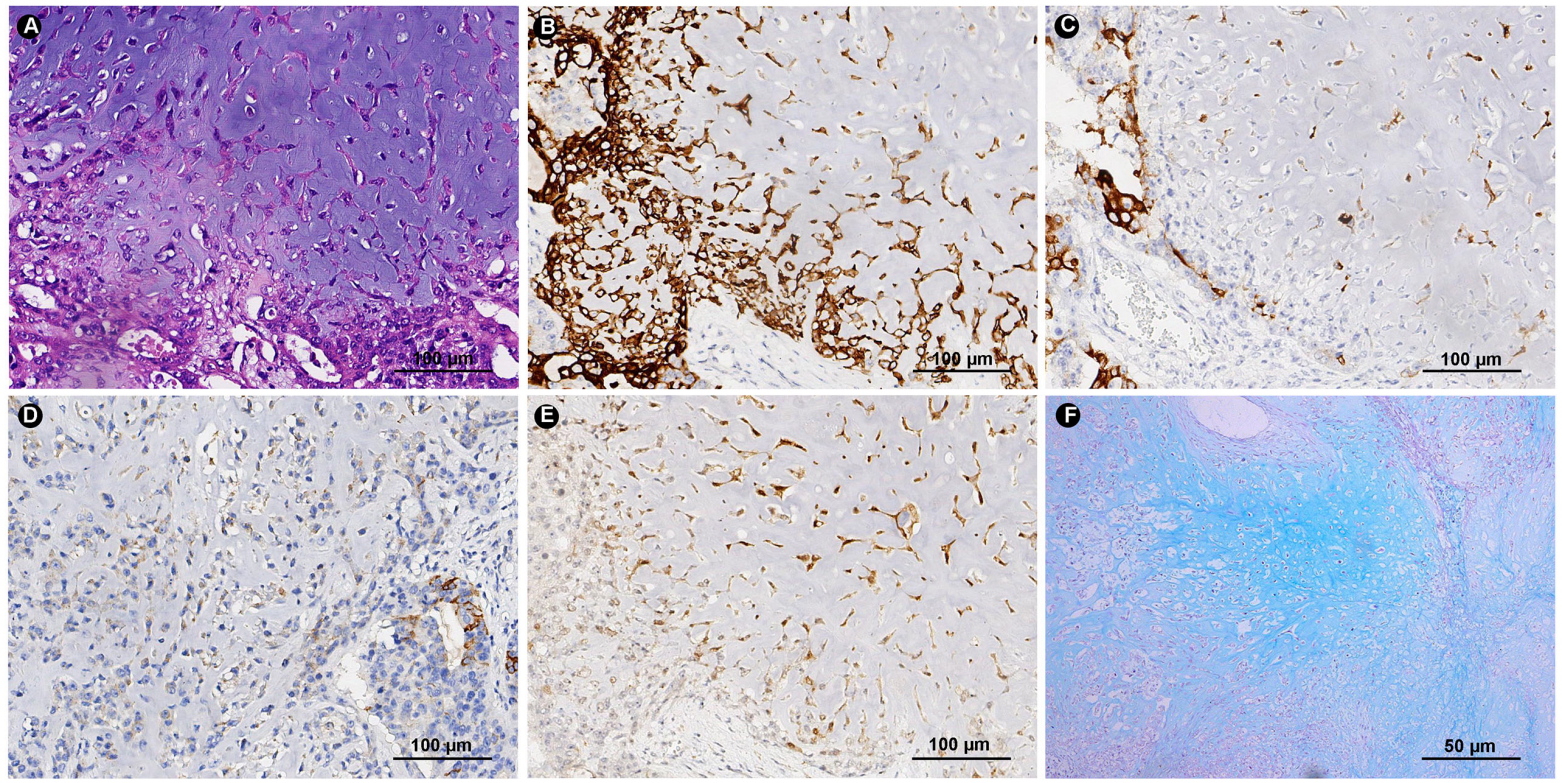
HE and Immunohistochemical. **(A)** The same site of HE section for immunohistochemical tissue sections (HE ×200). Tumor cells express CK7 **(B)**, EMA **(C)** MUC5AC **(D)** and S-100 **(E)** (IHC ×200). **(F)** Special histochemical staining of AB-PAS shows the abundant chondromyxoid matrix (Special stain ×100).

The adenocarcinoma cells expressed PCK, CK19, CK8/18, CK7, EMA and P53, showed focal positivity for S100, MUC5AC and MUC6, and were negative for AR. Tumor cells in the chondromyxoid matrix were positive for PCK, CK19, CK8/18, CK7 and S100 and negative for EMA, Calponin and P63. The Ki-67 index was approximately 50%. AB-PAS staining showed an abundant extracellular chondromyxoid matrix (**Figure 3**). In addition, the tumor cells were positive for MLH1, PMS2, MSH2 and MSH6 exhibiting microsatellite stability, and were negative for HER-2 and EBER.

The final diagnosis was poorly differentiated gastric carcinoma, partly poorly differentiated adenocarcinoma, and partly rich in chondromyxoid matrix, consistent with gastric carcinoma with chondromyxoid matrix similar matrix-producing metaplastic breast carcinoma. The tumor invaded the subserosa with vascular invasion and 7 out of 26 metastatic lymph nodes. After open total gastrectomy, no postoperative complications were found, and the patient was discharged after 2 weeks of hospitalization. Adjuvant chemotherapy was not accepted due to the patient’s personal wishes. The patient developed multiple liver metastases within 5 months postoperatively and died of the disease 7 months later.

## Discussion

In the 2019 WHO classification of digestive tumors, malignant epithelial tumors of the stomach are classified as adenocarcinoma, squamous cell carcinoma, adenosquamous carcinoma, undifferentiated carcinoma, gastroblastoma and neuroendocrine neoplasms. This report describes a case of gastric carcinoma with chondromyxoid matrix in an elderly male who has a large tumor with a well-defined at the stomach fundus and cardia. Microscopically, poorly differentiated adenocarcinoma is located on the surface of the tumor and arranged in solid and cribriform patterns. The chondromyxoid matrix is located inside the tumor with multinodular distribution. The adenocarcinoma area directly transitioned to the chondromyxoid matrix area (no intervening spindle cell sarcoma component). The characteristics of gastric carcinoma with a chondromyxoid matrix are different from those of gastric adenocarcinoma, and are also different from gastric carcinosarcoma reported so far. Gastric carcinosarcoma includes carcinoma and sarcoma components, among which sarcoma components have been reported to include chondrosarcoma (2, 3), osteosarcoma (4, 5), leiomyosarcoma (4), rhabdomyosarcoma (5) and sarcoma undifferentiated. Morphologically, this case is similar to gastric cancer with a chondrosarcoma component that has been reported, while different for immunohistochemistry (2). The chondrosarcomatous component is negative for cytokeratins (CK8/18) in the literature. However, tumor cells in the chondromyxoid matrix area diffusely expressed a variety of cytokeratins (PCK, CK19 CK8/18 and CK7), similar to the expression of adenocarcinoma in our case. It is possible that this case is another form of gastric carcinosarcoma.

Interestingly, this case has similar features to matrix-producing metaplastic breast carcinoma, both morphologically and immunohistochemically (6). As Wargotz et al (7) described matrix-producing metaplastic breast carcinoma is divided into two types: diffuse and peripheral. In the peripheral type, tumor cells are concentrated around the nodule and gradually decrease from the outside to the inside, while the chondromyxoid matrix gradually increases, and hemorrhage and necrosis are seen in the center of the tumor. In this case, the chondromyxoid nodules accounted for 80% of the entire tumor. The tumor cells around the nodules are abundant, and hemorrhage and necrosis are also seen in the center of the large nodules, similar to the peripheral type. In addition, the diffuse positive expression of S-100 is a characteristic marker of matrix-producing metaplastic breast cancer. In this case, tumor cells in the chondromyxoid matrix area are diffusely positive for S-100, PCK, CK19, CK8/18 and CK7, and the chondromyxoid matrix shows blue by AB-PAS staining. Therefore, based on morphological features and immunohistochemistry, we prefer to classify this case as gastric carcinoma with chondromyxoid matrix differentiation, which is not exactly equivalent to chondromyxoid sarcoma because the tumor cells in the chondromyxoid matrix area express a variety of keratins and may belong to a type of carcinosarcoma.

This case should be differentiated from gastric carcinosarcoma with chondrosarcoma, in which the chondrosarcoma component is focally expressed or negative for the epithelial markers CK and EMA, while the tumor cells in the chondromyxoid matrix area express a variety of keratins and EMA in this case. It should also be differentiated from metastatic matrix-producing metaplastic breast carcinoma, which has a history of breast cancer and the carcinoma component expresses GATA3, while this case does not.

In breast cancer, metaplastic breast cancer is more aggressive than the corresponding IBC-NST of the same age, stage and grade. Metaplastic carcinoma is characterized by a high stage, easy local recurrence and aggressive invasion, so the prognosis is poor. The prognosis of gastric adenocarcinoma is related to the degree of differentiation and the presence or absence of lymph node metastasis. In this case, the adenocarcinoma was moderately to poorly differentiated, and the chondromyxoid matrix component accounted for approximately 80%. Lymph node metastasis had already occurred at the time of diagnosis, and the metastatic component was adenocarcinoma. The patient developed multiple liver metastases 5 months postoperatively and died 7 months later. Although there was only one case, it also showed a poor prognosis, consistent with breast metaplastic carcinoma. In contrast to the treatment of gastric cancer, the treatment of breast cancer is mainly based on its molecular type, so it has no reference value. In this case, postoperative adjuvant chemotherapy is recommended, but the patient is unwilling to receive chemotherapy. He developed multiple liver metastases 5 months later and died of the disease 7 months after the operation, with a poor prognosis.

## Conclusion

We report this case because the unique morphological features cannot be classified into existing histological subtypes of gastric cancer. Based on morphological features and immunohistochemistry and referring to the concept of matrix-producing metaplastic breast cancer, we prefer to classify this case as gastric carcinoma with chondromyxoid matrix differentiation, which may belong to a novel morphology of carcinosarcoma.

## Data availability statement

The original contributions presented in the study are included in the article/supplementary material. Further inquiries can be directed to the corresponding author.

## Ethics statement

Written informed consent was obtained from the patients/participants for the publication of this case study.

## Author contributions

XW, HH, and JY conceived the study. XW wrote the first draft of the manuscript. XW LZ, and ZZ acquired data. All authors contributed to the article and approved the submitted version.
